# Komplikationen und deren Behandlung nach PAO

**DOI:** 10.1007/s00132-023-04359-5

**Published:** 2023-03-20

**Authors:** Lars Nonnenmacher, Alexander Zimmerer, André Hofer, Manuela Bohorc, Georg Matziolis, Georgi Wassilew

**Affiliations:** 1 Center for Orthopaedics, Trauma Surgery and Rehabilitation Medicine, Universitätsmedizin Greifswald, Ferdinand-Sauerbruch-Straße, 17475 Greifswald, Deutschland; 2 Deutsches Zentrum für Orthopädie, Waldkliniken Eisenberg, 07607, Eisenberg, Deutschland

**Keywords:** Periacetabuläre Ostetomie, Komplikationen, Hüftdysplasie, Korrektur, Risiko, Periacetabular Osteotomy, Complications, Hip dysplasia, Correction, Risk

## Abstract

**Hintergrund:**

Die Hüftdysplasie stellt die häufigste Ursache der sekundären Koxarthrose dar. Die periazetabuläre Osteotomie (PAO) nach Ganz ist ein etabliertes Therapieverfahren, welches eine reproduzierbare Korrektur der dreidimensionalen Pathologie erlaubt. Den mittel- und langfristigen guten Behandlungsergebnissen stehen potenzielle Komplikationsrisiken eines invasiven Beckeneingriffes gegenüber. In Anbetracht des vornehmlich jungen Alters der Patient*innen ist eine Kenntnis der möglichen Komplikationen und der daraus resultierenden adäquaten Therapie wichtig.

**Therapieentwicklung:**

Die kontinuierliche Weiterentwicklung der Operationstechnik und die zunehmende Erfahrung der Operateur*innen – mit dem sich hieraus ergebenden wachsenden Verständnis bezüglich kritischer Operationsschritte – haben zu einer wesentlichen Reduktion schwerwiegender Komplikationen geführt. Um darüber hinaus das Outcome für die Patient*innen zu verbessern, sind neben der Reduktion der Komplikationsrate auch ein besseres Verständnis bezüglich der hüftdysplasieassoziierten Begleitpathologien essenziell.

## Abstract (en)

**Background:**

Hip dysplasia is the most common cause of secondary hip osteoarthritis. The Ganz periacetabular osteotomy (PAO) is a well-established procedure that allows a reliable and reproducible correction of the complex pathology. The promising medium and long-term good treatment results are offset by the potential risk of complications from an invasive pelvic procedure. Considering the mainly young age of the patients, knowledge of the possible complications and the resulting adequate therapy is crucial.

**Treatment development:**

The continuous development of surgical techniques and increase in overall surgical experience alongside the appreciation of critical surgical steps have led to a substantial reduction of serious complications. In addition, to improve patient outcome, a greater understanding of the associated pathologies that may be related to hip dysplasia is essential.

Die periazetabuläre Osteotomie nach Ganz ist ein weltweit etabliertes Verfahren zur Behandlung der adulten Hüftdysplasie und azetabulären Retroversion. Die Kombination aus jungen und anspruchsvollen Patienten sowie einer gleichermaßen komplexen anatomischen Pathologie wie auch dreidimensionalen Korrekturmethoden erfordert eine hohe chirurgische Präzision. Das umfassende Verständnis möglicher Komplikationen und deren Therapieoptionen sind notwendig für eine adäquate Behandlung.

## Hintergrund

Seit der Erstbeschreibung der periazetabulären Osteotomie (PAO) durch Reinhold Ganz im Jahre 1988 wird eine rückläufige Rate schwerwiegender Komplikationen in der Literatur beschrieben [1]. Während Clohisy et al. [4] in einer Metaanalyse aus dem Jahre 2009 noch von einer Inzidenz schwerwiegender Komplikationen von 6–37 % berichteten, nennen aktuelle Studien Inzidenzen zwischen 4 und 7 % [1, 35]. Neben den schwerwiegenden Komplikationen werden in den aktuellen Studien leichtere Komplikationen in 11–14 % der Fälle beschrieben (Tab. 1). Es wird angenommen, dass die steigende Erfahrung mit der Operationsmethode und insbesondere die Reduktion der Zugangsmorbidität mittels minimal-invasiver Zugänge wesentlich zur Reduktion potenzieller Komplikationen beigetragen haben [1, 36]. Ein weiterer Vorteil der PAO gegenüber anderen Beckenosteotomien – wie beispielsweise der klassischen Tripleosteotomie nach Tönnis – ist eine relativ kurze Rekonvaleszenz. Aufgrund der geringeren Zugangsmorbidität, der kurzen Rekonvaleszenz und den vergleichsweise geringeren Komplikationsraten [27] hat sich die PAO mittlerweile international als Goldstandard in der Therapie der residuellen Hüftdysplasie etabliert. Aufgrund der zunehmenden Popularität der PAO und der flachen Lernkurve sind genaue Kenntnisse über die möglichen Komplikationen und deren Therapieoptionen unabdingbar [25].

**Table 1:** 

	Komplikation	Inzidenz (%)	95 %-KI
Schwergradig	Verletzung N. femoralis	0,9	0,1; 3,2
	Verletzung N. ischiadicus	1,5	0,6; 2,9
	Diskontinuität dorsale Säule	1,7	0,8; 3,1
	Intraartikuläre Osteotomie/Fraktur	3,1	1,4; 5,9
	Pseudarthrose	2,0	1,5; 2,5
	Thromboembolisches Ereignis	0,8	0,5; 1,2
	Überkorrektur	0,9	0,02; 4,9
	Unterkorrektur	1,3	0,3; 3,2
	HO Brooker-Stadien III + IV	1,0	0,7; 1,3
Leichtgradig	Verletzung NCFL	9,6	8,2; 11,1
	Wundhämatom	2,6	1,6; 4,0
	Implantatirritation	14,0	12,4; 15,7
	Wundheilungsstörung/Hüftgelenkinfektion	1,0	0,7; 1,5
	HO Brooker-Stadien I + II	19,2	16,2; 22,5

*HO* Heterotope Ossifikation, *NCFL* N. cutaneus femoris lateralis

Entsprechend aktueller Metaanalyse, Stand 11/22 [35]

## Wundheilungsstörungen/-infektionen

Seltene Komplikationen der PAO sind Wundheilungsstörungen oder Hüftgelenksinfektionen, welche mit einer Inzidenz von 1 % beschrieben werden [35]. Adipositas (BMI > 30 kg/m^2^) ist generell einer der Hauptrisikofaktoren für Komplikationen nach PAO [29] und unserer Erfahrung nach mit einem erhöhten Risiko insbesondere für die vorgenannte Komplikationsart assoziiert. Auch eine verlängerte Operationszeit gilt als Risikofaktor. Somit besteht eine direkte Korrelation zwischen der Erfahrung der Operateur*innen bzw. der Operationszeit und dem Auftreten von Infektionen [20]. Bei oberflächlichen Wundheilungsstörungen und Hüftgelenksinfektionen sollte umgehend ein chirurgisches Débridement, eine Lavage sowie ein Sekundärverschluss erfolgen. Bei einer Revisionsoperation des Hüftgelenkes sollte die Lavage z. B. mit Ringer-Lösung erfolgen. Eine Lavage mittels Antiseptika wie Polyhexanid, Wasserstoffperoxid oder Povidon-Jod muss vermieden werden, da diese Stoffe chondrotoxisch wirken können [28].

Beim Débridement einer tiefen Infektion ist ein Wechsel aller Schrauben sowie die komplette Entfernung von Fremdmaterial einschließlich der Nähte und Anker obligat. Zudem sollte unmittelbar postoperativ kalkuliert und im Verlauf resistenzgerecht antimikrobiell therapiert werden.

## Implantatirritationen

Der häufigste Grund für einen erneuten operativen Eingriff nach PAO stellt die symptomatische implantatassoziierte Irritation am Beckenkamm dar. Die Inzidenz liegt in der Literatur bei 14 % [35], tritt jedoch in unserer Kohorte deutlich häufiger auf. Insbesondere bei schlanken Patienten tritt gehäuft der Wunsch nach einer frühzeitigen Implantatentfernung auf, vor allem wenn die Schraubenköpfe nicht auf Knochenniveau versenkt werden konnten.

Eine weitere Indikation zur Implantatentfernung stellt die Reizung des M. iliacus dar, die insbesondere durch extraossär verlaufende Schrauben verursacht werden kann. Die Implantatentfernung führen wir nach röntgenologisch gesicherter knöcherner Konsolidierung ab dem 7. postoperativen Monat durch.

## Heterotope Ossifikationen

**Figure 1:**
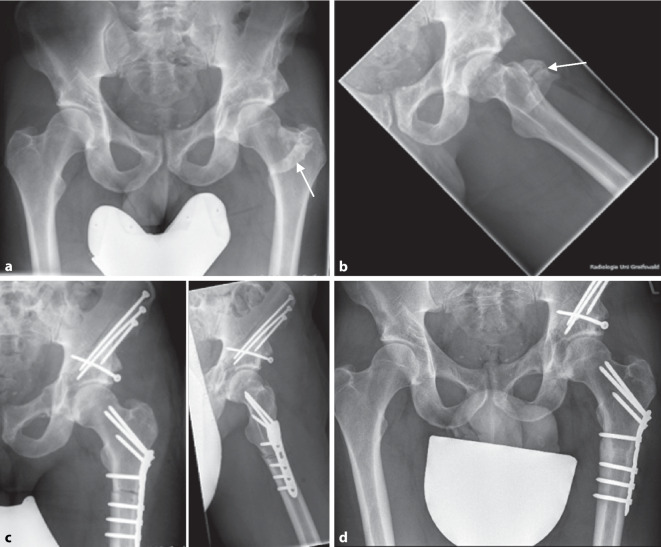

Heterotope Ossifikationen (HO) nach PAO werden in der aktuellen Literatur mit einer Inzidenz von 19 % für Brooker-Stadien I + II und mit 1 % für die schwerwiegenden Brooker-Stadien III + IV angegeben [35]. Der erste Schritt der Behandlung der Ossifikation ist deren Prävention. Wir empfehlen zur Ossifikationsprophylaxe die intraoperative Lavage und postoperativ die Einnahme von Coxiben oder alternativen nichtsteroidalen Antirheumatika für 21 Tage [23]. Eine zusätzlich durchgeführte Schenkelhalsmodulation könnte das Risiko für HO potenzieren. Eine zwingende Behandlungsindikation ergibt sich jedoch nur bei symptomatischen Ossifikationen, die eine Bewegungseinschränkung bedingen (Abb. 1).

## Nervenläsionen

Bei den Nervenläsionen muss zwischen den schwerwiegenden sensomotorischen Komplikationen durch Beeinträchtigung des N. femoralis, N. ischiadicus sowie N. obturatorius und den sensiblen Schäden des N. cutaneus femoris lateralis (NCFL) differenziert werden [13]. Die tägliche postoperative Verlaufskontrolle der peripheren Motorik und Sensibilität ist essenziell, da neurologische Defizite auch verzögert auftreten können [13, 16].

### Nervus cutaneus femoris lateralis

Die häufigste neurogene Komplikation betrifft die zugangsbedingte Schädigung des rein sensorischen NCFL. Hierbei tritt vornehmlich eine Hypästhesie im Bereich des lateralen Oberschenkels auf. Die postoperative Inzidenz wird in der Literatur mit großer Spannbreite von 1,5–74 % nach PAO beschrieben [2, 5]. Eine aktuelle Metaanalyse zeigte dabei eine durchschnittliche Inzidenz von 9,6 % [35]. Aus unserer Sicht ist diese Komplikation jedoch in der Literatur deutlich unterrepräsentiert. In einer vorangegangenen Studie ermittelte unsere Arbeitsgruppe eine postoperative Inzidenz von 51,3 % [36].

Unsere Beobachtungen wurden von Cates et al. in einer prospektiven Studie bestätigt, die bei 91 % der Patienten eine Hypästhesie im NCFL-Innervationsgebiet nach der Operation beobachteten und diese elektromyographisch objektivierten. Nach 3 Jahren präsentierten noch ca. zwei Drittel dieser Patienten persistierende Symptome im Sinne einer Hypästhesie, wobei sich die Symptomatik im Verlauf deutlich reduzierte [2]. In seltenen Fällen tritt jedoch eine Meralgia paraesthetica auf. Die betroffenen Patienten berichten von persistierenden Dysästhesien, häufig in Form eines kribbelnden oder brennenden Gefühls im Innervationsgebiets des NCFL. Ferner werden krampfartige Schmerzen im Oberschenkelbereich beschrieben [3]. Ist dieser Schmerz unter konservativer Therapie nicht regredient, sollte der Ursprung des neuropathischen Schmerzes differenziert werden [30]. Hierbei empfehlen wir die Infiltration mittels Lokalanästhetikum am Austritt des Nervs unter dem Leistenband aus der Lacuna musculorum (ca. 2 cm medial und kaudal der Spina ilica anterior superior). Stellt sich hierunter eine deutliche Schmerzreduktion ein, kann bei entsprechendem Leidensdruck eine Revisionsoperation mit einer Dekompression (Neurolyse) des NCFL erwogen werden. Tritt ein Rezidiv nach der Neurolyse durch eine erneute Vernarbung des Nervs auf, kann eine Neurektomie des Nervs mit einer Rückverlagerung in den Bauchraum durchgeführt werden [30]. Diese ist zwar mit einer permanenten Hypästhesie im zugehörigen Innervationsgebiet assoziiert, führt aber auch zu einer signifikanten Reduktion des Schmerzes [24].

### N. femoralis

Ein postoperativer Schaden des N. femoralis wird in der Literatur in 1 % der Fälle beschrieben [32, 35] und stellt nach unseren Beobachtungen eine noch seltenere Komplikation dar. Der N. femoralis unterliegt hauptsächlich der Gefahr eines Dehnungsschadens während der Osteotomie des Schambeins. Es ist daher bei der Präparation unbedingt darauf zu achten, dass ein schmaler Hohmann-Haken unter den M. iliopsoas auf das Schambein gesetzt wird, da dieser die natürliche Grenze zum N. femoralis darstellt. Um den N. femoralis während der Darstellung des Schambeinastes auf größtmögliche Distanz zur Osteotomie und zum Haken zu bringen sowie den M. iliopsoas zu entspannen, sollte das Bein flektiert, adduziert und innenrotiert werden [13, 36]. Ein weiteres Risiko für einen femoralen Nervendehnungsschaden besteht bei der azetabulären Reorientierung. Insbesondere bei ausgeprägter präoperativer Retroversion besteht die Gefahr, dass eine notwendige antevertierende Medialisierung (> 2 cm) des azetabulären Fragments in einer Überdehnung des N. femoralis resultiert [13]. Ein weiterer intraoperativer Fallstrick ist ein großer pubischer Osteotomiespalt, in dem neben der Psoas-Sehne auch der N. femoralis eingeklemmt werden kann. Sollte postoperativ ein funktionelles Defizit in Form motorischer Kraftminderung der aktiven Hüftbeugung und Knieextension und/oder sensorische Ausfälle im Bereich des anteromedialen Ober- sowie Unterschenkels auftreten, so sind diese hinweisend auf eine Läsion des N. femoralis [18]. Der erste Schritt des diagnostischen Algorithmus beginnt in solchen Fällen mit einer fachneurologischen Evaluation, gefolgt von einer weiterführenden Diagnostik mittels Nervenleitgeschwindigkeit und Elektromyographie sowie einer entsprechenden Bildgebung (Röntgen, MRT). Weil sich eine ausgesprochen gute Rekonvaleszenz unter konservativer Therapie in einer beschriebenen Zeitspanne von 3–12 Monaten zeigt, ist nur bei hochgradigem Verdacht einer Einklemmung in den Osteotomiespalt die chirurgische Exploration des N. femoralis indiziert [32].

### N. ischiadicus

Verletzungen des N. ischiadicus werden in ca. 1,5 % der Fälle beschrieben [35]. Im Gegensatz zum N. femoralis verläuft der N. ischiadicus knöchern geschützt im Bereich der dorsalen Säule, sodass weniger die Gefahr eines Dehnungsschadens als einer direkten akzidentiellen Schädigung bei Durchführung der Osteotomien besteht. Ein erhöhtes Risiko besteht im Rahmen der unvollständigen Sitzbeinosteotomie, falls es hier zu einer Durchtrennung der dorsalen Säule oder einer unkontrollierten lateralen Perforation kommt. Es wird daher empfohlen, die Osteotomie unter wiederholter Bildwandlerkontrolle am medialen Rand des Sitzbeines zu beginnen. Das Osteotom sollte dabei zur kontralateralen Schulter ausgerichtet werden. Im Gegensatz zum N. femoralis führt eine Flexion und Adduktion im Hüftgelenk dazu, dass der N. ischiadicus gespannt und näher an das Os ischii geführt wird [13]. Eine vollständige Extension und zusätzliche Abduktion im Hüftgelenk reduziert dementsprechend die Spannung auf den N. ischiadicus. Deswegen sollte das Bein vor Beginn der lateralen Sitzbeinosteotomie vollständig extendiert und leicht abduziert werden [36]. Darüber hinaus besteht ein relatives Risiko der Verletzung des N. ischiadicus im Rahmen der supra- sowie retroazetabulären Osteotomien. Dies einerseits direkt durch eine versehentliche Unterbrechung der dorsalen Säule und andererseits durch forcierte Mobilisation des Fragments, insbesondere im Falle einer unvollständigen Osteotomie mit noch bestehenden knöchernen Brücken [16]. Falls postoperativ sensomotorische Defizite auftreten, die auf eine Läsion des N. ischiadicus hinweisen, empfehlen wir eine umgehende radiologische Evaluation. Im Gegensatz zu Läsionen des Femoralis-Nervs zeigen solche des Ischiadicus-Nervs ein deutlich schlechteres konservatives Regenerationspotenzial mit häufig persistierenden Einschränkungen, sodass die operative Revision mit einer Neurolyse großzügig indiziert werden sollte [32].

Vornehmlich im angloamerikanischen Raum werden Fallserien mit intraoperativem Neuromonitoring publiziert [22, 33]. Diese Methode hat sich aufgrund des deutlichen zeitlichen Mehraufwandes, der hohen Kosten und des fraglichen Nutzens bisher nicht etabliert. So können zwar intraoperativ bei adäquater Elektrodenlage Manöver detektiert werden, die ursächlich für einen Nervenschaden sein könnten. Die postoperative Auftretenswahrscheinlichkeit konnte dadurch aber nicht signifikant gesenkt werden [13]. Gleichzeitig besteht bei unsachgemäßer Elektrodenableitung die Gefahr einer trügerischen Sicherheit.

## Knöcherne Komplikationen

### Pseudarthrosen

Eine verzögerte knöcherne Konsolidierung bzw. Pseudarthrose nach PAO kommt in rund 2 % der Fälle vor [31, 35]. Das Risiko einer Pseudarthrose korreliert mit dem Alter, dem BMI und dem Schweregrad der Dysplasie. Selberg et al. untersuchten bei 245 Patienten die zu erwartende Zeitspanne bis zur vollständigen knöchernen Konsolidierung der Osteotomien. Nach 6 Monate wurde erst bei 45 % und nach 12 Monaten bei 92 % der Patient*innen eine vollständige knöcherne Konsolidierung beobachtet [31]. Symptomatisch waren dabei Patienten mit einer Pseudarthrose der hinteren Säule, welche dann im Verlauf eine Revisionsoperation benötigten. Die weiteren Pseudarthrosen des Os pubis und des Os ischii waren asymptomatisch [31].

### Frakturen

Während der Osteotomien besteht das Risiko für unerwünschte Osteotomieverläufe, Frakturen sowie Fissuren. Die Gesamtinzidenz ossärer Läsionen im Rahmen der PAO beträgt dabei in der Literatur 3,1 % [35].

Am häufigsten treten in das Azetabulum ziehende Fissuren auf

Am häufigsten treten hierbei in das Azetabulum ziehende Fissuren auf, die bei der retroazetabulären Osteotomie entstehen können. Diese Fissuren heilen meist ohne Anpassung der Nachbehandlung aus. Komplexe Azetabulumfrakturen aufgrund von intraoperativ erkannten intraartikulären Osteotomien müssen gegebenenfalls reponiert werden und ohne Korrektur der Dysplasie ausheilen. Die intraoperative Fraktur der hinteren Säule kann sowohl bei der Sitzbein- als auch bei der retroazetabulären Osteotomie entstehen. Diesbezüglich konnten Morris et al. [19] zeigen, dass bei Nutzung eines breiten Meißels alternativ zum Ganz-Osteotom das Risiko signifikant steigt. Aufgrund des erhöhten Risikos einer symptomatischen Pseudartrose, empfehlen wir eine prolongierte Teilbelastung und eine regelmäßige radiologische Kontrolle (6, 12 und 18 Wochen postoperativ).

Wird eine Fraktur oder Fissur der hinteren Säule intra- oder direkt postoperativ nicht erkannt, kann eine Insuffizienzfraktur des unteren Schambeinastes entstehen [17]. Diese kann in der 6‑wöchigen postoperativen Kontrolle apparent werden. Diesbezüglich sollte ebenfalls eine prolongierte Teilbelastung durchgeführt werden. Wir raten den Patienten in diesem Fall von einem Übergang in schmerzadaptierte Vollbelastung ab und empfehlen stattdessen, für weitere 6 Wochen im 3‑Punkt-Gang mit 50 % des Körpergewichtes zu belasten. Im Anschluss daran erfolgt eine erneute radiologische Kontrolle und bei adäquater Kallusbildung der Übergang in die Vollbelastung. Findet keine Konsolidierung sowohl der hinteren Säule als auch des unteren Schambeinastes statt, sollte bei weiterer Dislokation eine Revision erwogen werden. Ist im postoperativen Verlauf keine weitere Dislokation darzustellen, kann zugewartet werden. Die Indikation zur Revision sollte erst nach 9–12 Monaten gestellt werden. Hierbei spielt die CT eine entscheidende Rolle. Klinisch beschreiben diese Patienten häufig belastungsabhängige Schmerzen in der Scham- und der dorsalen Glutealregion sowie im Adduktorenbereich.

**Figure 2:**
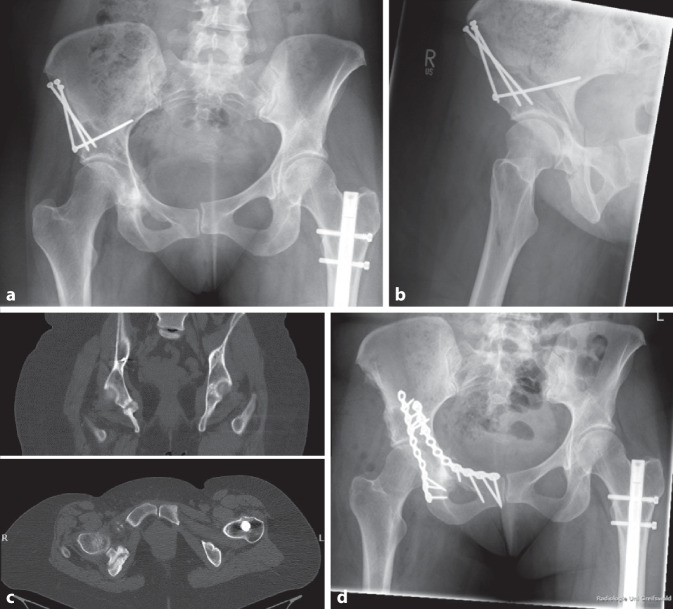

Im Rahmen der Revisionsoperation erfolgt eine Anfrischung der Pseudarthrose und es wird eine dorsale Plattenosteosynthese durchgeführt. Häufig ist eine Pseudarthrose der hinteren Säule ebenfalls mit einer Pseudarthrose des Os pubis assoziiert. Daher sollte bei der Revision auch der vordere Pfeiler mit einer vorderen Plattenosteosynthese adressiert werden (Abb. 2). Dabei kann autogener Knochen aus dem Ilium gewonnen werden, um knöcherne Defekte aufzufüllen.

**Figure 3:**
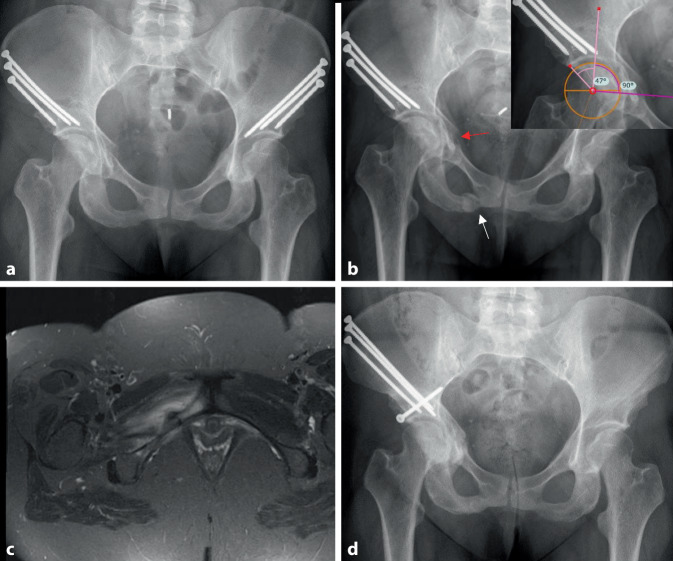

Postoperative Stressfrakturen stellen in der allgemeinen Literatur eine unterrepräsentierte Komplikation dar und werden in spezifischen Studien mit einer Inzidenz von 15,4 % [19] respektive 18,4 % [17] angegeben. Besteht in der Röntgenuntersuchung ein Verdacht, kann eine Schnittbildgebung (Abb. 3) sinnvoll sein. Eine Restriktion der Mobilisierung und eine Verlängerung der Teilbelastung führt in 91 % der Fälle zu einer Konsolidierung von Stressfrakturen [17].

Eine schlechte Knochenqualität kann zu einer Iliumfraktur durch das voreilige Repositionsmanöver mittels Steinmann-Pin bei noch nicht liberiertem azetabulärem Fragment führen. Ebenso können die Pins dabei aus dem Fragment ausreißen. In diesen Fällen ist das Umsetzen der Steinmann-Pins notwendig, um eine suffiziente Korrektur erreichen zu können. Um das Risiko für diese Komplikation zu minimieren und – falls diese eintreten sollte – genügend Knochen für das Umsetzen der Steinmann-Pins zur Verfügung zu haben, sollte die supraazetabuläre Osteotomie möglichst 3 cm über dem kranialen Pfannenrand durchgeführt werden [36].

Beim Einbringen der Schrauben können Frakturen der Crista iliaca vornehmlich im anterioren Anteil auftreten. Diese sind durch eine Umplatzierung der Schrauben ausreichend adressiert. So kann auf eine horizontale Schraube durch das azetabulären Fragment in das Ilium oder durch eine dorsale Schraubenposition an der Crista iliaca konvertiert werden. Aus unserer Sicht sind in der Regel drei Schrauben notwendig. Es können entweder drei durch das Ilium ins azetabuläre Fragment oder zwei Iliumschrauben mit einer horizontalen Schraube kombiniert werden.

## Vaskuläre Komplikationen

### Gefäßläsionen und Blutungsereignisse

Gefäßverletzungen und massive Blutungsereignisse sind in Anbetracht der Invasivität und eingeschränkten Visualisierbarkeit einiger Operationsschritte mit einer Inzidenz von 0,5 % eine relativ selten auftretende Komplikation [35]. Das größte Risiko einer direkten Gefäßverletzung besteht im Rahmen der Schambeinosteotomie. Die in der Nähe verlaufenden (neuro-)vaskulären Obturatorstrukturen sowie die häufig vorhandene Corona mortis müssen durch eine subperiostale Präparation und Positionierung stumpfer Retraktoren konsequent geschützt werden [36]. Die Corona mortis, eine meist venöse Gefäßanastomose zwischen der A. obturatoria und A. iliaca externa oder A. epigastrica inferior, liegt bei ca. 75 % der Patienten vor und verläuft anteromedial der Eminentia iliopubica [11]. Die Schambeinosteotomie sollte daher medial der Eminentia iliopubica immer streng subperiostal und nur im Schutz der Retraktoren bei gebeugtem sowie adduziertem Hüftgelenk erfolgen. Auch wenn orthopädische Fallbeschreibungen von pelvinen Gefäßverletzungen nach PAO selten sind, so unterstreichen Fallberichte aus der Gynäkologie, Allgemein- und Unfallchirurgie die hämodynamisch relevante Blutungsgefahr bei Verletzung dieser Strukturen [11].

Weitere potenzielle Blutungsquellen stellen die intraossären Äste der A. iliolumbalis, die azetabulären Äste der A. glutea superior und inferior sowie die A. circumflexa femoris lateralis dar. Die knochenversorgenden Äste der A. iliolumbalis sind im Rahmen der proximalen subperiostalen Präparation des M. iliacus und der supraazetabulären Osteotomie gefährdet. Ganz erwähnte diese häufige Blutungsquelle und die gelegentliche Notwendigkeit von Knochenwachs zur Blutstillung bereits in seiner Erstbeschreibung [9]. Kommt es zu einer intraoperativ stärkeren Blutung empfehlen wir, zunächst den mittleren Blutdruck des Patienten zu kontrollieren und den Bereich zu tamponieren. Ist der Blutdruck eingestellt und besteht immer noch eine verstärkte Blutung, die gegebenenfalls hämodynamisch relevant ist, sollte unverzüglich ein Gefäßchirurg dazu gerufen werden oder – sofern dies nicht möglich ist – der Patient in die Radiologie zur Intervention verlegt werden. Diese Gefäßverletzungen müssen intraoperativ nicht apparent sein. Daher sollte bei einem postoperativen Blutdruckabfall mit Anstieg der Herzfrequenz ein hämorrhagisches Geschehen differenzialdiagnostisch immer in Betracht gezogen werden. Bei subklinischem Verlauf empfehlen wir eine zügige Bildgebung mittels Kontrastmittel-CT. Bei rasch progredientem Verlauf mit hypovolämischen Schockzeichen empfiehlt sich die notfallmäßige Angiographie mit Interventionsbereitschaft.

**Figure 4:**
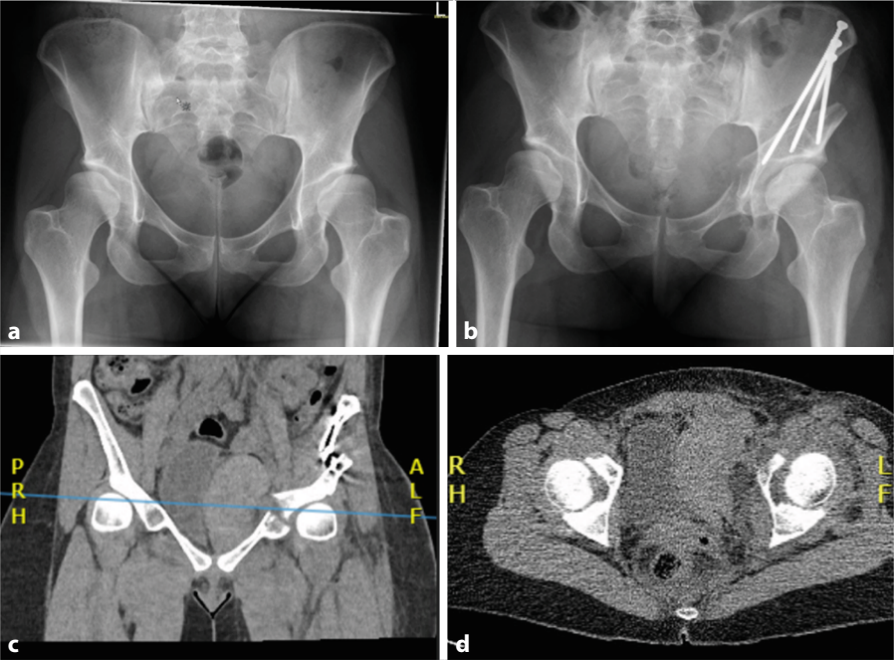

Abb. 4 zeigt einen Fall, bei dem postoperativ abdominelle Beschwerden und eine Dysurie auftraten. In der CT zeigte sich ein großes, retroperitoneales Hämatom als Hinweis auf eine stattgehabte Blutung. Hämatome treten in 2,6 % der Fälle auf [35].

### Thromboembolische Ereignisse

Die PAO wird als Beckeneingriff gemäß der aktuellen S3-Leitlinie „Prophylaxe der venösen Thromboembolie“ der Hochrisikogruppe zugeordnet [8]. Weitere thromboembolische Risikofaktoren sind die postoperative Teilbelastung, das prädominante weibliche Geschlecht sowie eine damit häufig assoziierte Kontrazeption. Die Wahrscheinlichkeit eines thromboembolischen Ereignisses wird mit 0,8 % angegeben [35]. Eine Thromboseprophylaxe mit niedermolekularen Heparinen oder adäquaten Alternativen ist bis zur sicheren Vollbelastung empfohlen. Im Falle eines thromboembolischen Ereignisses sollte unverzüglich eine leitliniengerechte Therapie erfolgen.

## Muskuläre Komplikationen

Muskuläre Komplikationen nach PAO finden in der gängigen Literatur keine Erwähnung. Während Ganz et al. 1988 noch das Absetzen diverser Muskelansätze beschrieb, ist durch eine stetige Optimierung gegenwärtig der operative Zugangsweg mit verschiedenen minimal-invasiven Techniken möglich [9, 36]. Bei der gängigsten minimal-invasiven Technik ist das Absetzen des M. sartorius an der Spina iliaca anterior notwendig. Eine suffiziente Refixation ist essenziell, um einen postoperativen Ausriss bei Mobilisation zu verhindern. In früheren Beschreibungen wurde ein größeres knöchernes Fragment herausgelöst, um dieses suffizient mittels Schraube zu refixieren. Ein Ausriss dieses Fragments und die häufig durchgeführte Reosteosynthese gestaltete sich jedoch schwierig. Deswegen ist man dazu übergegangen, den Ansatz des M. sartorius durch eine sehr schmale Osteotomie der Spina iliaca anterior superior abzulösen und mittels Fadencerclage zu refixieren [14]. Eine Limitation der aktiven Flexion des Hüftgelenks für 6 postoperative Wochen wird dabei als notwendig betrachtet.

**Figure 5:**
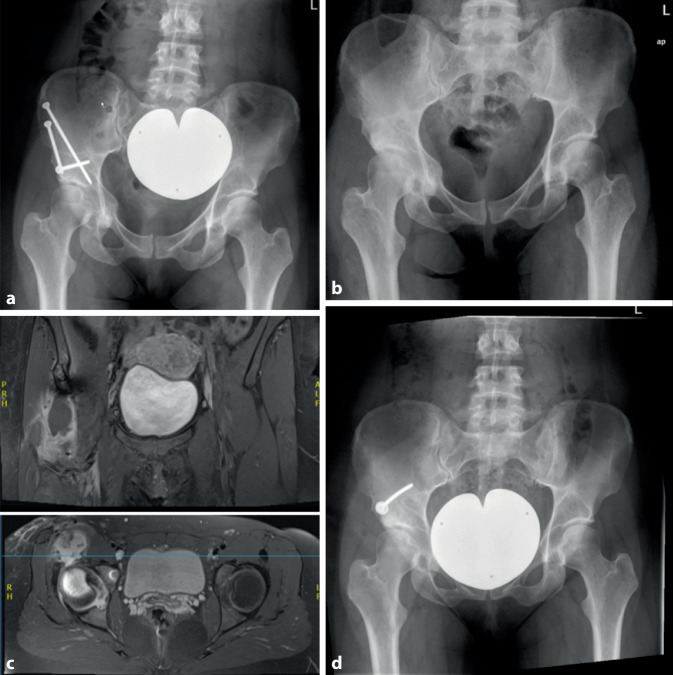

Die Ablösung des M. rectus femoris entspricht nicht der aktuellen Definition einer minimal-invasiven PAO. Bei Ablösung und Refixation besteht das Risiko einer Ruptur des Sehnenansatzes des M. rectus femoris (Abb. 5.). Bei hinreichendem Verdacht ist eine weiterführende Diagnostik mittels MRT zur Diagnosesicherung indiziert. Eine Refixation kann erwogen werden, wenn ein funktionelles muskuläres Defizit entsteht, welches nicht kompensiert werden kann, wie beispielsweise bei aktiven Sportlern. Präventiv sollte insbesondere bei Patienten mit einem hohen sportlichen Anspruch eine präoperative Aufklärung bezüglich dieser Komplikationen bei vorzeitiger Belastungssteigerung erfolgen. Ungeachtet dessen ist die physiotherapeutische Beübung für eine adäquate Rekonvaleszenz nach PAO von entscheidender Bedeutung. Persistierende Kraftdefizite nach PAO sind nicht selten. So zeigte eine dänische Studie, dass 12 Monate nach PAO weiterhin muskuläre Defizite vorliegen können und insbesondere die Fähigkeit der Hüftflexion sowie -abduktion mit der postoperativen Zufriedenheit korreliert [12].

## Beschwerdepersistenz

Bezüglich der Langzeitergebnisse nach PAO sind 20-Jahres-Überlebensraten ohne Konversion auf ein künstliches Hüftgelenk bei einem Arthrose Grad 0 und I von 81 % respektive 65 % beschrieben [34, 37]. Diese Ergebnisse spiegeln jedoch nicht differenziert das Outcome und die Zufriedenheit der Patienten nach PAO wider. So ist ein Teil der Patienten nach PAO weiterhin unzufrieden und berichtet über persistierende Hüftbeschwerden. Die Restbeschwerden werden in der Literatur mit einer Inzidenz von 7–27 % beschrieben [10, 15].

Es sollte in der Nachuntersuchung eine adäquate Differenzierung zwischen intraartikulären und extraartikulären Pathologien erfolgen. Schmerzen nach PAO können neben technisch suboptimalen Korrekturergebnissen (Über- oder Unterkorrektur) auch durch einen Weichteilkonflikt, Pseudarthrosen, Meralgia paraesthetica (siehe oben), Psoas-Pathologien, Labrumläsionen, chondrale Defekte, femoroazetabuläre Impingementsyndrome (FAI), femorale Torsionsfehler etc. bedingt sein. Im ersten Schritt muss klinisch und radiologisch als Grund zunächst eine Unter- oder Überkorrektur ausgeschlossen werden. Ein entscheidender Faktor ist dabei die postoperative Beweglichkeit des Hüftgelenks als Hinweis auf ein FAI. Dieses FAI kann neben einer anterolateralen Überkorrektur des azetabulären Fragments auch durch ein nicht behandeltes Cam und/oder eine Coxa magna und/oder eine femorale Torsionspathologie oder durch einen vorangeschrittenen Verschleiß des Hüftgelenks bedingt sein. Weitere Tests wie der Flexion-Adduktion-Innenrotation-, der Flexion-Abduktion-Außenrotation- sowie der Abduktion-Hyperextension-Außenrotation-Test und Psoas-Test, welche für ein FAI, eine chondrolabrale Pathologie oder eine Psoas-Irritation richtungsweisend sein können, sind bei der Untersuchung obligat. Zur radiologischen Standarddiagnostik gehören eine Beckenübersicht a.-p., eine 45°-Dunn- sowie eine Faux-profile-Aufnahme. Führt diese Standardbildgebung nicht zur Diagnose, kann eine Schnittbildgebung mittels MRT und CT notwendig sein.

### Intraartikuläre Pathologien

Eine weitere Ursache für Residualbeschwerden können begleitende chondrolabrale Pathologien sein, die durch eine alleinige PAO nicht direkt adressiert werden konnten [6, 7, 26]. Häufig führt eine weiterführende Diagnostik mittels MRT aufgrund des einliegenden Osteosynthesematerials zu keiner eindeutigen Diagnose. Hier kann eine Infiltration zur Differenzierung zwischen extra- und intraartikulären Pathologien hilfreich sein. Mögliche intraartikuläre Pathologien beinhalten Labrum- und Knorpelläsionen, Adhäsionen sowie eine Ruptur des Lig. capitis femoris.

Sind intraartikuläre Pathologien nicht auszuschließen, kann bei der Schraubenentfernung eine begleitende arthroskopische Diagnostik des peripheren und des zentralen Kompartiments oder nach der Schraubenentfernung eine MRT durchgeführt werden. Dass eine verbleibende Asphärizität mit einem schlechteren Langzeitüberleben assoziiert ist, konnte ebenfalls gezeigt werden [37].

Insgesamt bestehen jedoch bezüglich der assoziierten Pathologien, wie chondrolabrale Schäden, nur wenig Evidenz, ob die Adressierung nach PAO zur Verbesserung des Outcomes führt [15].

### Technisch suboptimale Operationsergebnisse

**Figure 6:**
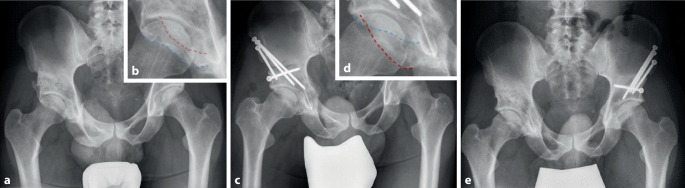

Die Indikationen zur Revision nach vorangegangener PAO können bei klinisch symptomatischer Überkorrektur mit einem resultierenden FAI oder bei Unterkorrektur mit persistierenden Instabilitätssymptomen gestellt werden. Die radiologische Unterkorrektur tritt dabei mit 20 % zehnfach häufiger auf als eine Überkorrektur (2 %; Abb. 3; [21]). Die Auftretenswahrscheinlichkeit einer Unterkorrektur korreliert mit der präoperativen Schwere der Dysplasie und den anatomischen Merkmalen der Dysplasie (kurze Knorpeltragfläche). Eine Überkorrektur kann zu einem sekundären Impingement im Sinne einer Pincer-Morphologie führen. Auch die adäquate azetabuläre Anteversion ist für ein zufriedenstellendes Operationsergebnis entscheidend. Abb. 6 zeigt den Fall einer ausgeprägten postoperativen Retroversion, die einer Revisions-PAO zugeführt wurde.

## Ausblick

Die rasante Weiterentwicklung der 1988 beschriebenen PAO nach Ganz hat bereits zu einer deutlichen Reduktion schwerwiegender Komplikationen geführt. In den kommenden Jahren wird der Fokus auf einem genaueren Verständnis relevanter Begleitpathologien liegen. Es gilt herauszufinden, welche dieser Pathologien das Behandlungsergebnis entscheidend beeinflussen und zu welchem Zeitpunkt diese vorzugsweise therapiert werden sollten. Ein weiterer wesentlicher Aspekt wird die verbesserte Ausbildung junger Operateure sein, um die beschriebene erhöhte Komplikationsgefahr zu minimieren. Aktuelle Trends wie Kadaverkurse und Fellowships bei erfahrenen „High-volume“-Operateuren werden unabdingbar sein. Innovative Technologien wie z. B. patientenindividuelle Schnittblöcke, roboterassistierte Navigation oder Augmented Reality könnten bei der PAO ebenfalls von Nutzen sein.

## Fazit für die Praxis

- Die periazetabuläre Osteotomie ist eine weltweit etablierte Behandlungsmethode der residuellen Hüftdysplasie, die mit einer Vielzahl unterschiedlicher Komplikationen einhergehen kann.
- Die Kenntnis möglicher Komplikationen ist für das behandelnde Team essenziell, um sowohl präventive als auch im Falle einer Komplikation adäquate therapeutische Maßnahmen einleiten zu können.
- Assoziierte Komplikationen umfassen u. a. Verletzungen von Nerven, Gefäßen, Knochen und Muskeln, heterotope Ossifikationen, Wundheilungsstörungen, Wundinfektionen, Implantatirritationen sowie thromboembolische Ereignisse.
- Intraoperative Prophylaxe vor Schädigung N. femoralis: Flexion + Adduktion + Innenrotation im Hüftgelenk.
- Intraoperative Prophylaxe vor Schädigung N. ischiadicus: Extension + Abduktion im Hüftgelenk.
- Dem Eingriff muss eine detaillierte Diagnostik und Aufklärung vorangehen, in der die Risiken genannt und eingeordnet werden. Ebenso muss auf eine mögliche Beschwerdepersistenz hingewiesen werden.

## Abkürzungen

BMI: Body-Mass-Index
FAI: Femoroazetabuläres Impingementsyndrom
HO: Heterotope Ossifikation
NCFL: N. cutaneus femoris lateralis
PAO: Periazetabuläre Osteotomie
